# Solid lipid nanoparticles for increased oral bioavailability of acalabrutinib in chronic lymphocytic leukaemia

**DOI:** 10.1186/s11671-024-04157-8

**Published:** 2024-12-30

**Authors:** Swagata Sinha, Punna Rao Ravi, Makarand Somvanshi, S. R. Rashmi

**Affiliations:** Department of Pharmacy, Birla Institute of Technology and Science Pilani, BITS-Pilani Hyderabad Campus, Jawahar Nagar, Kapra Mandal, Medchal District, Telangana, 500078 India

**Keywords:** Tyrosine kinase inhibitor, Solvent-free hot emulsification, Biphasic release systems, Spleen distribution, Lymphatic uptake

## Abstract

Acalabrutinib (ACP) is a first-line treatment for chronic lymphocytic leukemia but suffers from poor and variable oral bioavailability due to its pH-dependent solubility, CYP3A4 metabolism, and P-gp efflux. Thus, the objective of this study was to improve the solubility and dissolution behaviour, in turn enhancing bioavailability, by formulating solid lipid nanoparticles (SLNs). ACP loaded SLNs (ACP-SLNs) were prepared via solvent-free hot emulsification followed by a double sonication process. A combination of glyceryl di-behenate and stearyl palmitate along with Tween 80 was used as the lipid phase to dissolve ACP. A 1% w/v Poloxomer188 solution served as the aqueous phase. The optimized ACP-SLNs were spherical in shape and had particle size of 234.7–257.5 nm, PDI of 0.261–0.320 and loading efficiency of 18.70 ± 1.78%. A typical biphasic release pattern was observed from ACP-SLNs in the in vitro dissolution studies under gastrointestinal and plasma pH conditions (> 90% drug release at pH 4.5 ± 0.2, 6.8 ± 0.2 (representing GIT), and 7.4 ± 0.2 (representing plasma) at 8, 16 and 24 h, respectively). The freeze-dried product was stable when stored at 5 °C for over 6 months. Compared with the bulk drug suspension, the ACP-SLNs suspension resulted in 2.29-fold increase in oral bioavailability and more importantly 2.46-fold increase in the distribution of drug to spleen. Additionally, inhibition of lymph production and flow by administering cycloheximide resulted in 46.01% decrease in the overall absorption of ACP-SLNs, indicating the significance of lymphatic uptake process in the oral absorption of ACP-SLNs.

**Graphical Abstract:**

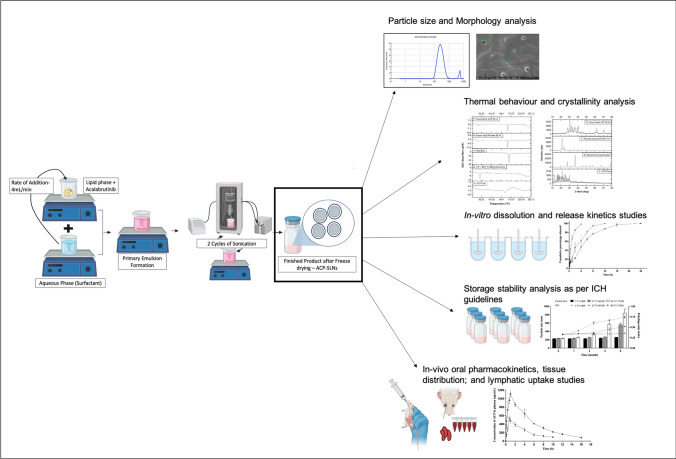

## Introduction

Chronic lymphocytic leukaemia (CLL) is a slow-progressing heterogeneous neoplastic disorder characterized by abnormal B lymphocyte proliferation (> 5,000 cells per μL of blood) and is usually diagnosed around the age of 55–65 years in humans [1]. The development of CLL, like other cancers, primarily depends on the microenvironment consisting of cellular elements, generation of new vessels, availability of growth factors, immunogenic protection of neoplastic cells, etc.. The T cells, stromal cells, and macrophages fuel the proliferation and survival of neoplastic cells. A previous study revealed that tyrosine kinases, specifically the B-cell receptor (BCR) pathway, play a significant role in the development of CLL and maintenance of its microenvironment via various antigen interactions [2–4]. In addition to conventional therapies with chlorambucil, bendamustine, and fludarabine, which have several severe side effects, various tyrosine kinase inhibitors (TKis), such as ibrutinib, acalabrutinib, venetoclax, and dasatinib, provide targeted therapy and improved tolerance [4–6]. In the entire cascade of tyrosine kinases in the B-cell receptor (BCR) axis, Bruton's tyrosine kinase (BTK) is a key target, whose activation leads to consecutive phosphorylation and activation of other kinase pathways. Thus, molecules such as ibrutinib, acalabrutinib, bosutinib, and zanubrutinib, which specifically inhibit the phosphorylation of BTK, are the first-line therapies [7, 8]. BTK inhibitors (BTKis) share similar overall structural patterns, with highly hydrophobic substituent groups such as aromatic amines, heterocyclic aromatics, and biaryl groups [9, 10]. Thus, most BTKIs belong to either BCS class II (low solubility and high permeability) or BCS class IV (low solubility and low permeability).

Acalabrutinib (ACP), a type of selective irreversible BTKi, belongs to BCS class II. It is marketed as Calquence, 100 mg capsules and tablets (active ingredient in the form of acalabrutinib maleate), by AstraZeneca, Cambridge, UK [11, 12]. The molecule shows pH-dependent solubility- ACP is practically insoluble above pH 6 [11, 12]. Zhou D. et. Al, 2022, reported that, the dynamic gastrointestinal pH influences the pH at the surface which ultimately affect the solubility of ACP (a weakly basic compound having a maximum basic pKa at 5.77) [13]. The gradual increase in the pH from 1 to 7, perilously impacted both C_max_ and AUC ultimately resulting in an oral bioavailability of 25 ± 11%. The low and variable bioavailability is also the result of its extensive metabolism by CYP3A enzymes and efflux by P-gp systems in the small intestine [11, 12, 14]. The metabolism of the drug was also assessed as part of its elimination profile, revealing that unchanged ACP accounted for less than 2% of the total amount eliminated (84% excreted in faeces and 12% in urine) [12, 14]. Thus, the formulation of a nanoparticulate drug delivery system may increase the bioavailability of the drug not only by direct uptake of the formulation into the systemic circulation, circumventing the need for drug dissolution in small intestinal lumenal fluids but also by preventing the exposure of the drug to P-gp and CYP3A enzymes in enterocytes. A population PK study by Edlund H. et. Al, 2018, revealed that ACP showed a two compartmental oral pharmacokinetic model- a fast absorption phase (T_max_ = 0.5 to 0.75 h) followed by a bi-exponential elimination phase (a rapid decline followed by a slower phase) [15]. Lipid-based drug delivery systems such as solid lipid nanoparticles (SLNs), composed of a solid core (0.1 to 30% w/w; made of either triglycerides, fatty acids, or wax alone or in combination) coated with an amphiphilic stabilizer or surfactant (0.5 to 5% w/w; such as poloxamers, lecithin, or polysorbates) [16], may offer an improved delivery of ACP. The lipophilic core, which is solid at both room temperature and physiological temperature, enhances drug loading and facilitates controlled release of the entrapped lipophilic drug. Additionally, the presence of lipids affects the gastric emptying time, allowing the formulation to be present for a longer duration. The interaction between SLNs and enterocytes could lead to increased chylomicron production, facilitating the uptake of the SLN lipid matrix through lymphatic drainage [17, 18]. The suitability and increased chances of uptake of SLNs by the lymphatic system was well proven and reported by Ravi P. et. Al, 2014 for BCS class II molecules like lopinavir and raloxifene [19, 20]. The formulation of SLNs consists of three major steps: the formation of a primary O/W nanoemulsion or microemulsion, reducing the particle size, achieving uniform dispersity via the application of any high-energy mechanical force, and finally, the solidification of the molten lipid to form distinct particles. The formation of the primary emulsion can be carried out without or with any organic solvent. However, for techniques involving an organic solvent, efficient removal of the added solvent is of utmost importance, as it can be toxic to the body. The required high energy is provided by processes such as homogenization (high shear or high pressure) and ultrasonication.

However, till date a very few attempts have been taken for the improvement of oral bioavailability of ACP by the virtue of nanoparticle based drug delivery. Recent research from our group has demonstrated that formulating ACP as nanocrystals significantly enhances its dissolution rate- over 85% of ACP dissolved within 60 min, compared to the free base [21]. Additionally, the absolute oral bioavailability of ACP was improved to 34.15% ± 9.015% in Wistar rats (22.83% ± 5.015% for the free base). Secondly, Shettiwar A. et. Al, 2024 developed a linalool based orally administrable system offering 5.1-fold increase in oral bioavailability in Balb/c mice [22].

Hence, the current work emphasizes the formulation of novel lipid based nano delivery systems, ACP-SLNs, via a solvent-free hot emulsification technique followed by a double sonication process. The optimized formulation was evaluated for its physical characteristics*, *in vitro release of ACP at different pH values, and storage stability. Furthermore, the impact of ACP-SLNs on the oral bioavailability of ACP in comparison with that of conventional aqueous suspensions of ACP was evaluated via in vivo pharmacokinetic studies in male Wistar rats. Additionally, the extent of the influence of lymphatic uptake on the oral absorption of ACP-SLNs was studied in a suitable in vivo model.

## Materials

### Chemicals and animals

Acalabrutinib (ACP) and prednisone (internal standard, IS) was a gift sample from MSN Laboratories Pvt. Ltd., Hyderabad, India; and Strides Pharma Pvt. Ltd., Hyderabad, India, respectively. Cycloheximide (CHX) (98% pure) was obtained from Loba Chemie Pvt. Ltd., Mumbai, India. Glyceryl di-behenate (GB) (Compritol 888 ATO) was obtained as a gift from Gattefosse India Pvt. Ltd., Mumbai, India and stearyl palmitate (SP) (Kolliwax® S), Poloxomer 188 (P188) (Kolliphor® 188 Geismar), and Tween 80 (T80) (Kolliphor® PS 80) were obtained from BASF-India Pvt. Ltd., Mumbai, India. All other reagents, such as salts for buffer and solvents, were procured from Sisco Research Laboratories (SRL) Pvt. Ltd., Mumbai, India. For all the experiments, water was collected from a Milli-Q water purification system (Millipore, MA, USA).

Male Wistar rats (weighing approximately 220–250 g) were procured from VAB Life Sciences, Hyderabad, India (282/PO/RcBt/S/2000/CPCSEA), after thorough evaluation and approval of the animal study protocol by the institutional animal ethics committee (IAEC).

### Instruments and software

For the preparation of SLNs, a magnetic stirrer (RCT Basic, IKA India Private Limited, Bangaluru, India), a high-sheer homogenizer (Polytron PT 3100 D stand dispenser fitted with PT-DA 12 / 2EC-F154 dispensing aggregate, Kinematica AG, Malters, Switzerland) and ultrasonic processing equipment (Sonics Vibracell VCX 750 attached with a 13 mm solid standard probe) were used. The particle size, polydispersity index and zeta potential were determined via a zeta sizer (Zeta sizer NANO ZS, Malvern Pananalytical Ltd., Worcestershire, UK). The SLNs were freeze dried via a freeze dryer (Coolsafe 110–4, LaboGene A/S, Allerød, Denmark). For in vitro dissolution studies, a USP type II dissolution tester (TDT-08 L) with a temperature controlling unit (ETC-11 L) (Electrolab India Pvt. Ltd., Mumbai, India) was used. A Shimadzu Prominence HPLC system equipped with a degasser unit (DGU-20A3R), two pumps (LC-20AD), an autosampler unit (SIL-20ACHT), a column housing unit with a temperature controller (CTO-20AC), and a photodiode array (PDA) ultraviolet (UV) detector system (SPD-M20A) (Shimadzu Corporation, Kyoto, Japan) was used for all the chromatographic analyses. A vacuum concentrator (Scan Vac) connected to a condenser (Coolsafe 110–4, LaboGene A/S, Allerød, Denmark) was used for processing biomatrix-based samples and for freeze-drying the SLN dispersion. Dissolution modelling and analyses of in vivo pharmacokinetic data were performed via Phoenix WinNonlin software (Version 8.4.0.6172, Pharsight Corporation, NC, USA).

## Experimental methods

### Preparation of ACP-loaded Solid Lipid Nanoparticles (ACP-SLNs)

#### Screening of formulation components

The screening of the excipients, viz. lipids and stabilizers (stabilizers in both the internal and external phases), was based on various critical factors, namely, the solubility of ACP in the excipients; the particle size and polydispersity index (PDI) of the SLNs; and the stability of the SLNs at 2–8 °C and 25 ± 2 °C.

##### Selection of solid lipid(s)

The solid lipids were screened on the basis of biocompatibility and visual evaluation of the solubility of ACP in the lipids. Solid lipids such as GB (Compritol® 888 ATO), glyceryl di-stearate (Precirol ATO 5), glyceryl trimyristate (Dynasan 114), stearic acid, SP (Kolliwax® S) and cetyl palmitate were selected for solubility studies, as they were reported to be biocompatible. In the solubility studies, approximately 200 mg of each lipid was weighed separately and melted by heating to 5–10 °C above its corresponding melting point using a magnetic stirrer equipped with a temperature controlling program. ACP was added in small amounts to each lipid (in molten state), incrementally, up to a maximum of 100 mg under continuous stirring (150–200 rpm) while maintaining the temperature (5–10 °C above its corresponding melting point) until some amount of ACP was present in undissolved / powder form in the lipid. Further, the lipids in which the drug has similar solubility, the lipid solutions (ACP dissolved in lipid) were maintained at the same temperature for 60 min to observed for any precipitation of the drug.

##### Selection of surfactant(s)

Surfactants are primarily chosen on the basis of two criteria: firstly, the affinity of ACP towards the aqueous solution in the presence of the surfactant; and secondly, their capacity to form a stable emulsion when the solid lipid is combined with the aqueous phase. Thus, the solubilitysolubilities of ACP was assessed individually in 0.15 % w/v and 2% w/v solutions of different surfactants such as P188, poloxamer 407 (P407), T80, polyvinyl alcohol (PVA), polyvinyl pyrrolidone K30 (PVP K30), Gelucire 48/16, and Gelucire 44/14. Further, on the basis of the solubility of ACP in various surfactants, separate placebo batches of SLNs were prepared using the selected surfactant(s), in order to assess their ability to stabilize the SLNs by evaluating the particle size via dynamic light scattering.

#### Preparation of ACP-SLNs

ACP-SLNs were prepared via previously used method of solvent-free hot melt emulsification [19] modified by adding subsequent process of double ultrasonication for size reduction. A 250 mg lipid mixture containing GB and SP in a 3:7 ratio (75 mg GB and 175 mg SP) was placed in a beaker and melted at 75 °C. To the molten lipid, T80 (1% w/w of the total solid mass which includes the solid lipids and drug) was added under stirring at 150 rpm while maintaining the temperature at 75 °C. To the above mixture, 40 mg of ACP powder was gradually added under the aforementioned conditions until a clear solution was obtained. An aqueous solution of P188 (1% w/v; 50 mL of solution per batch of formulation) was prepared in a separate container and heated to 75 °C. The P188 solution was added to the lipid phase at a rate of 4 mL/min under a stirring rate of 1200 rpm while maintaining the temperature at 75 °C. The stirring rate and temperature were maintained for 15 min following the complete addition of the aqueous phase to the lipid phase to obtain a primary emulsion. The primary emulsion was immediately subjected to probe ultrasonication at 25% amplitude and 55 °C for 10 min in pulse mode (with 10 s on and 05 s off). The formulation was subjected to a second or final cycle of sonication (25% amplitude, 25 °C, 4 min with a 10 s on and 5 s off pulse rate) after cooling to 25 ± 2 °C under a stirring rate of 250 rpm.

To freeze dry the resultant SLNs, the freshly prepared batch was centrifuged at 16,627 $\times$ g for 30 min to form a pellet. The pellet was washed thoroughly two times with 0.5% w/v P188, 10 mL followed by centrifugation at 16,627 g$\times$ for 30 min using a table top cooling centrifuge (5430R; Eppendorf, Hamburg, Germany). The final pellet obtained was reconstituted with the same solution of P188 (0.5% w/v; 10 mL volume used), followed by freeze drying using 2% w/v mannitol as a cryoprotectant.

### Chromatographic conditions

To quantify the loading and entrapment efficiency of the ACP-SLNs and the in vitro dissolution study samples of the formulations, a high-performance liquid chromatography (HPLC) method using a Phenomenex Luna^Ⓡ^ Omega polar C-18 column (150 mm × 4.6 mm, 5 µm) maintained at 55 °C was developed and validated as per the latest ICH guidelines. The mobile phase was composed of 48 parts of 10 mM ammonium acetate (pH 3.5) with 52 parts methanol. The flow rate was 1.05 mL/min, with a 50 µL injection volume and a total runtime of 7 min in the analysis of each sample. A photodiode array ultraviolet detector (PDA-UV) set at a wavelength of 250 nm was used in the analysis. The retention time for ACP was 4.24 ± 0.02 min. The calibration range was linear (0.05 to 5 µg/mL), with limits of detection and quantification of 1.46 ng/mL and 4.32 ng/mL, respectively.

To determine the concentration of ACP in samples prepared from biomatrices (plasma or spleen samples collected in in vivo pharmacokinetic and tissue distribution studies), the validated HPLC–UV-based method developed by the current research group was used [21]. The mobile phase was composed of 60 parts of 10 mM ammonium acetate (pH 3.5) with 40 parts methanol using the same column mentioned above. The biomatrices were processed via a single-step protein precipitation method using acidified (1% v/v formic acid) methanol at a ratio of 1:8 (plasma:precipitant) to extract the drug as well as the internal standard (prednisone). The extracted supernatant was evaporated via a vacuum concentrator (1200 rpm, 15 °C for 2.5 h) and further reconstituted with the mobile phase for quantification. The column was maintained at 53 °C with the mobile phase pumped at a flow rate of 1.1 mL/min and a 50 µL sample injection volume. The total runtime was 12 min for each sample, with a detection wavelength of 250 nm. ACP eluted at a retention time of 5.96 ± 0.03 min, whereas prednisone eluted at 8.29 ± 0.02 min.

### Characterization studies

#### Physical evaluation—particle size, PDI, zeta potential, and morphology

During the formulation development and optimization trials, the particle size and PDI of the SLNs were evaluated via a zeta sizer through principles of dynamic light scattering. For the optimized formulation, both freshly prepared and reconstituted freeze-dried powder (2 mg/mL powder in filtered Milli-Q water, dispersed uniformly by vortex mixing (Vortex 3, IKA India Pvt. Ltd., Bengaluru, India) for 5 min set at the speed mark of 3 (approximately 1000 rpm)) were diluted (1000 $\times$ dilution with filtered Milli-Q water) and analysed via pre-set measurement parameters, including a sample equilibration time of 120 s in the sample compartment, a measurement temperature of 25 °C, a detector fixed at a backscatter angle of 173°, and an average response of 15 iterations/runs for each sample. The surface charge or zeta potential of the samples were measured using folded capillary zeta cell. The measurements were done using the same zeta sizer instrument based on the Smoluchowski model built in the software (Zeta sizer ver. 8.02) with the Debye function (F(κa)) value set at 1.50.

Morphological features such as shape and surface pattern were evaluated via field emission scanning electron microscopy (FE-SEM) (Apreo LoVac, FEI, Oregon, USA) equipped with a Leica EM ACE200 vacuum sputter coater, Leica microsystems GmbH, Wetzlar, Germany. The samples were prepared via the drop-casting technique, where approximately 50–75 μL of liquid sample was placed on a clean silicon wafer stuck to an aluminum stub. The sample was allowed to vacuum dry at 25 °C overnight, followed by gold and aluminium combination sputter coating (approximate thickness of the 10 nm coating layer) under an argon gas atmosphere. Images were captured via microscopic evaluation at different magnifications via an xT microscope ver. 13.5.0 software.

#### Thermal analysis via DSC

The thermal properties of pure ACP, a physical mixture of SP and GB (1:1), and the freeze-dried blank SLNs as well as ACP-SLNs were analysed via DSC-60 (TA-60 WS, Shimadzu, Kyoto, Japan). Approximately 4 mg samples, with the exception of pure ACP, were weighed in aluminum pans designed for DSC, crimp sealed, and placed in the instrument sample chamber for equilibration at 30 °C. For pure ACP, a weight equivalent to the amount of ACP loaded in 4 mg of lyophilized ACP-SLNs (18.5% of 4 mg = 0.74 mg) was used in the analysis. The thermograms were recorded in the temperature range of 30–300 °C at a controlled heating rate of 5 °C/min under nitrogen purging (50 mL/min). The thermograms obtained were analysed via TA-60 software.

#### Powder X-ray diffraction (P-XRD)

Pure ACP (18.5% of 15 mg = 2.78 mg), physical mixture of 7 parts of SP with 3 parts of GB, and ACP-SLNs (15 mg freeze dried powder) was subjected to P-XRD analysis using a ULTIMA IV instrument by Rigaku, MA, USA with a Scintillation counter detector. The measurements were conducted in 2θ mode over a range of 10 to 80 degrees using a copper X-ray source, scanning at 3 degrees per minute, and maintaining a temperature of 25 °C. The characteristic peaks were analysed using PDXL2 software.

#### Loading and encapsulation efficiency of ACP-SLNs

The loading and entrapment efficiency (%LE and %EE) of the freshly prepared formulations were determined via centrifugal filter devices equipped with an Ultracel® low-binding regenerated cellulose membrane with a 10,000 molecular weight cut-off (MWCO) (Amicon® Ultra 10 K device, Millipore, Merck KGaA, Darmstadt, Germany). A total of 2000 μL of freshly prepared sample was placed in the filter device and subjected to centrifugation at 14,167 × g at 15 °C for 15 min. The filtrate containing the free drug was analysed via the HPLC‒UV analytical method described in Sect. 3.2, and the %LE and %EE values of the ACP-SLNs were calculated via the following formulas (Eqs. (1) and (2)).

1 $$\% Loading\;Efficiency \left( {\% LE} \right) = \left( {\frac{{Total\;amt_{ACP} - Amt_{free ACP} }}{{Total\;amt\;of\;solids_{ACP + excipients} }}} \right) \times 100$$

2 $$\% Entrapment\;Efficiency \left( {\% EE} \right) = \left( {\frac{{Total\;amt_{ACP} - Amt_{free ACP} }}{{Total\;amt_{ACP} }}} \right) \times 100$$

where $Total\;amt_{ACP}$ represents the amount of ACP added per batch (40 mg for a batch of 50 mL); $Amt_{free ACP}$ represents the amount of free ACP quantified in the filtrate; and $Total\;amt\;of\;solids_{ACP + excipients}$ represents the total mass of the solids in a batch, which includes both the amount of lipids and the drug.

#### In vitro dissolution studies of ACP-SLNs

Dissolution studies were performed using USP type II dissolution apparatus under conditions simulating the fluids in different parts of the gastrointestinal tract (200 mL of pH 1.2 ± 0.02; 4.5 ± 0.02 buffer; and 900 mL of pH 6.8 ± 0.02 buffer) and blood/plasma (500 mL of pH 7.4 ± 0.02 buffer). Bulk ACP and freeze-dried ACP-SLNs powder (both equivalent to 100 mg of ACP) were dispersed in dissolution media containing 0.5% w/v sodium dodecyl sulfate (SDS). The temperature was maintained at 37 ± 2 °C with a constant stirring rate of 75 rpm. At predetermined time intervals, 1000 μL samples were withdrawn from the dissolution media via a syringe and replenished with the same volume of fresh media (to retain the sink condition). The samples were immediately centrifuged at 15,373 × g in an ultracentrifuge. The supernatant was collected, diluted with mobile phase (methanol with 10 mM (pH 3.5) ammonium acetate buffer at a ratio of 52:48), and analysed via an HPLC–UV-based analytical method (mentioned in Sect. 3.2). The dissolution data obtained were contoured into different mathematical models (zero order; first order; and Higuchi square root kinetics) to determine the kinetics of drug release. The best fit model was analysed on the basis of the regression coefficient.

#### Stability studies of ACP-SLNs

As per the guidelines provided by the International Conference on Harmonization (ICH) Q1A(R2), the freeze-dried ACP-SLNs powder (n = 3 under each condition) was stowed in clear glass vials, sealed, and placed in 3 different stability chambers (Mack Pharmatech Pvt. Ltd., Mumbai, India) maintaining 45 ± 2 °C/75 ± 5% RH; 25 ± 2 °C/60 ± 5% RH; and 5 ± 2 °C over a period of 6 months. The stability of the samples was analysed on the basis of three parameters: average particle size, PDI, and content uniformity. In addition, at the end of 6 months, the drug dissolution of the most stable sample set was assessed at pH 7.4 ± 0.02 per the protocol for analysis described in Sect. 3.3.4, and the results were compared against those of the freshly prepared formulation. The deviations in dissolution were calculated in terms of the similarity factor (f2) via equation no. 3 [23]. As per the US FDA CDER guidelines, the f2 value for two similar formulations should be ≥ 50%.

3 $$Similarity\;factor \left( {f2} \right) = 50 \times Log \left\{ {\left[ {1 + \left( {{\raise0.7ex\hbox{$1$} \!\mathord{\left/ {\vphantom {1 n}}\right.\kern-0pt} \!\lower0.7ex\hbox{$n$}}} \right)\mathop \sum \limits_{t = 1}^{n} R_{t} - T_{t} } \right]^{ - 0.5} \times 100} \right\}$$

where $n$ represents the number of time points; $R_{t}$ represents the dissolution value of the pre-change batch/the freshly prepared ACP-SLNs; and $T_{t}$ represents the dissolution value of the post-change batch/freeze-dried ACP-SLNs stored under specific conditions after 6 months.

### Haemolytic assay to assess the toxicity of ACP-SLNs

Red blood cells (RBCs) were isolated from blood drawn from healthy Wistar rats and then formulated into a 2% v/v suspension using saline solution (0.9% w/v NaCl solution). Freeze-dried ACP-SLNs were reconstituted with filtered Milli-Q water to yield 1, 2.5, 5, 7.5 and 10 mg/mL ACP. The reconstitution was performed by dispersing the freeze-dried powder in Milli-Q and vortex mixing for 5 min set at the speed mark of 3 (approximately 1000 rpm) to ensure uniformity. A volume of 100 μL from each of the above suspensions was externally spiked into 900 μL RBC suspension to yield final concentrations of 0.1, 0.25, 0.5, 0.75 and 1.0 mg/mL of ACP, which were subsequently incubated for 1 h at 37 ± 2 °C. Saline and 1% v/v Triton X were used as negative (indicating 0% haemolysis) and positive (indicating 100% haemolysis) controls, respectively. After 1 h of incubation, all the samples were centrifuged at 3500 rpm for 10 min. The supernatant was analysed via a UV‒visible spectrophotometer at 545 nm. The extent of haemolysis was calculated via equation no. 4.

4 $$\% Haemolysis = \frac{{Abs_{sample} - Abs_{ - ve control} }}{{Abs_{ + ve control} - Abs_{ - ve control} }} \times 100$$

where $Abs_{sample}$ represents the absorbance values of samples containing ACP; $Abs_{ - ve control}$ represents samples spiked with saline; and $Abs_{ + ve control}$ represents samples treated with 1% v/v Triton X-100 at 545 nm.

Before performing the actual in vivo pharmacokinetic studies, an ACP-SLNs suspension (reconstituted aqueous suspension of freeze-dried ACP-SLNs powder by dispersing it in Milli-Q and vortex mixing for 5 min set at the speed mark of 3 (approximately 1000 rpm) followed by sonication for 2 min to ensure uniformity and easy administration.) was administered at a drug dose of 30 mg/kg through the oral route in Wistar rats to assess the safety of the formulation. The morphology of the RBCs separated from the blood samples collected at the time the maximum drug concentration in the blood (T_max_) (as per the bulk ACP suspension) was reached was examined and compared against the positive (1% v/v Triton X) and negative (saline solution) controls described earlier. The RBCs were separated from the blood by centrifugation (3500 rpm, 10 min, 4 °C) and suspended in cold phosphate buffer solution (pH 7.4) to yield a 2% v/v RBC suspension. To lyse the RBCs (positive control), 1% v/v Triton X was used. The RBCs, both intact and lysed, were fixed with 2% v/v glutaraldehyde solution and incubated at 4 °C for 1 h in the dark to ensure effective fixation. The excess glutaraldehyde was removed by washing the cells with cold PBS for 3 cycles. The cells were then dehydrated by allowing for 15 min each in 30%, 50%, 80%, 90%, and 100% v/v ethanol, followed by hydration in cold Milli-Q water. A very small quantity of the final suspension was drop cast on a P-type silicon wafer and allowed to gradually dry at 20 ± 2 °C. The dried samples were sputter coated (10 nm thickness) with a combination of gold (80%) and aluminum (20%), followed by SEM analysis at different magnifications.

### In vivo pharmacokinetic studies of ACP-SLNs

The in vivo pharmacokinetic study protocol (BITS-Hyd/IAEC/2021/15) was approved by the IAEC prior to the study. The animals were procured and quarantined for 15 days before any treatment was administered under constant environmental conditions (12 h light/dark cycle at 22 ± 1 °C at room temperature and 50 ± 10% relative humidity). The animals were provided access to food and water and acclimatized to the housing conditions. Prior to dosing, the animals were labelled, grouped and shifted to clean cages overnight fasting (10–12 h) with free access to water only. This not only prevents drug‒food interactions but also prevents coprophagia. This condition was continued until 4 h postdosing. After 4 h of dosing, the rat chow diet was allowed ad libitum. Healthy male Wistar rats (weighing approximately 250 g) were used in all the in vivo studies.

#### Intravenous bolus and oral pharmacokinetic studies

Pharmacokinetic studies were performed by administering ACP solution through the intravenous route as a bolus (at a drug dose of 12 mg/kg) and aqueous suspensions of bulk ACP and freeze-dried ACP-SLNs through the oral route (both at a drug dose of 30 mg/kg). The ACP solution was freshly prepared by dissolving the drug in a suitable solvent system (n-methyl pyrrolidone, 0.25% Tween 80, and water for injection at a volume ratio of 1:1:3) and administered at a dosing volume of 3 mL/kg through the tail vein via a syringe. An aqueous suspension of bulk ACP was prepared by dispersing the drug powder in distilled water. The freeze-dried ACP-SLNs powder (containing ACP equivalent to 30 mg/kg) was dispersed in distilled water (4 mL/kg dosing volume), vortexed, sonicated to form a uniform nanosuspension, and administered via oral gavage.

Blood samples (0.3 mL) were collected at various time points (predose, 0.88, 1.67, 0.42, 0.75, 1, 2, 4, 6,8, 10, 12, and 24 h) from the retro-orbital plexus into tubes containing 4% w/v di-sodium EDTA solution (10% v/v) for both intravenous and oral pharmacokinetic studies. These samples were then processed and analysed via the HPLC–PDA UV bioanalytical method discussed in Sect. 3.2. The time versus plasma concentration of ACP data obtained from each animal were fit into a suitable compartmental model via Phoenix WinNonlin software ver. 8.4.0.6172, and the intravenous and oral pharmacokinetic parameters were determined for ACP.

#### Spleen distribution study

The distribution of ACP to the spleen (the target organ) following the oral administration of aqueous suspensions of ACP and nanosuspensions of ACP-SLNs in Wistar rats (n = 4 for each treatment) was studied. At two specific time points, one at 0.75 h (which is the T_max_ of the aqueous suspension of ACP) and at 3.75 h (which corresponds to T_max_ + 2 × t_1/2_ of the drug in the rats), the animals that received the treatments were euthanized, and the whole spleen was collected from each animal. Any remnant adipose tissues attached to the spleen were removed. The spleen tissues were then washed with cold PBS (pH 7.4), patted dry, and weighed. Each spleen tissue sample was minced and homogenized separately at 7300–8000 rpm via a tissue homogenizer (T10 basic ULTRA-TURRAX® attached to an 8 mm probe; IKA India Private Limited, Mumbai, India) after the addition of ice-cold filtered PBS (3 mL per gram of tissue) to form a homogenate. To 100 μL of tissue homogenate, 10 μL of IS (2.5 μg/mL) was added and mixed, followed by the addition of 800 μL of acidified methanol to precipitate the proteins and extract ACP and IS from the homogenate. After thorough mixing, the processed homogenate samples were centrifuged at 14,167 × g and 8–10 °C for 12 min. The clear supernatant (~ 890 μL) was collected and concentrated via a ScanVac vacuum concentrator (at a centrifugation speed of 1200 rpm and 10 °C for 4 h). The dried concentrate was reconstituted with 100 μL of mobile phase (methanol and ammonium acetate buffer (pH 3.5) at a 40:60 ratio as used in the bioanalytical method), mixed and centrifuged (at a centrifugation speed of 14,167 × g, 8–10 °C for 10 min). The clear supernatant was analysed via a validated HPLC–PDA UV bioanalytical method, as described in Sect. 3.2.

### Role of lymphatic transport in the oral absorption of ACP-SLNs

In this study, male Wistar rats (n = 6, weighing approximately 250 g) were randomly distributed into two groups (n = 3 in each group) after 8–10 h of fasting. One group was administered saline (control group; dosing volume of 4 mL/kg through the intraperitoneal route), whereas the other group was administered cycloheximide (at a dose of 2 mg/kg and a dosing volume of 3.2 mL/kg through the intraperitoneal route). One hour after the administration of saline or cycloheximide, a nanosuspension of ACP-SLNs (at a drug dose of 30 mg/kg) was administered orally to the two groups. Blood samples were collected from the two groups at predose, 0.88, 1.67, 0.42, 0.75, 1, 2, 4, 6,8, 10, 12, and 24 h after the nanosuspension of ACP-SLNs was administered. In the present study, the animals were not provided food even after the 4th h of dosing to eliminate any chances of drug‒food interactions or adverse effects. Both water and saline solution were provided ad libitum. The blood samples were processed and analysed via the HPLC–PDA UV bioanalytical method according to the process described in Sect. 3.2.

### Statistical analysis

The data obtained from the experiments are reported as the mean ± standard deviation except for the time to reach the maximum concentration (T_max_) in the pharmacokinetic studies, which is reported as the median. Data obtained from comparative studies involving 2 treatment or formulation groups were analysed via a t test, considering equal variances, at a statistical significance level of 5%.

## Results and discussion

### Preparation of ACP-SLNs

#### Selection and effects of formulation components

##### Selection of solid lipid(s)

**Fig. 1:**
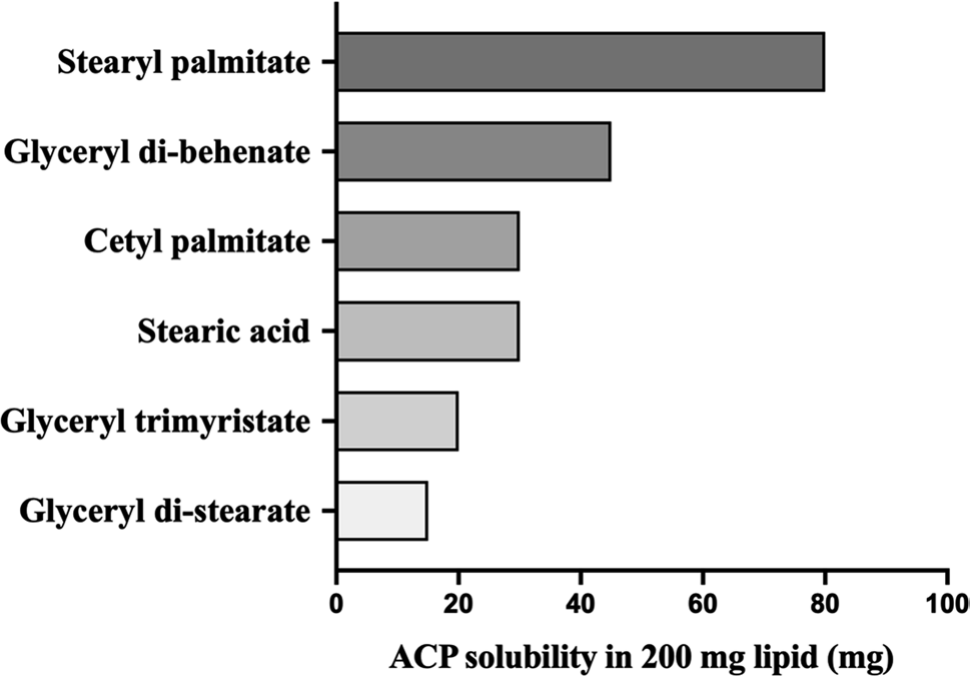
Extent of solubility of ACP in different lipids (~ 200 mg)

The selection of a suitable solid lipid (either a single or combination of two or more lipids) is critical for designing SLNs. For the preparation of ACP-SLNs, various biocompatible solid lipids were screened from the larger pool on the basis of their suitability for oral formulation, their ability to carry the maximum amount of the drug dose and their melting point range of 65 ± 10 °C. Thus, the solubility of ACP was investigated in 200 mg of selected orally administrable, biocompatible solid lipids. Among these, the maximum solubility of ACP was observed in SP, followed by GB, without any traces of precipitation (Fig. 1). Additionally, with increase in solubility of ACP in the lipid, the colour of the solution (ACP dissolved in molten lipid) changed from pale pink to a dark scarlet.

##### Selection of surfactant(s)

Apart from the selection of lipids, screening of surfactants is also important and is based on several critical factors, such as the extent of solubility of ACP in the surfactant solution; the dispersibility of the lipid melts in the aqueous phase; the particle size and PDI; the stability of the formulated SLNs in the dispersion after cooling or congelation of the lipids; and the %LE [24, 25]. Table 1 shows that ACP was least soluble in P188, followed by PVA and T80. This ensures that in the presence of either of these three surfactants, ACP will have the least affinity towards the aqueous phase and will be contained in the lipid phase, ensuring better drug loading, particularly during the manufacturing process of the ACP-SLNs. Furthermore, on the basis of the trials performed (Batch No. BF4‒S25, F5‒S25, and F6‒S25, Table 2), P188 was chosen as the surfactant on the basis of its average particle size and particle size distribution and stability at 2‒8 °C. A literature survey revealed that P188 exhibits thermos-reversible behaviour, which may impact the viscosity of the aqueous phase and, in turn, the particle size reduction process or the process of congelation when kept at lower temperatures. However, when 0.5 to 1.2% w/v P188 was used in the aqueous phase, negligible alterations in viscosity were observed. Thus, the effect of P188 on reducing the particle size and maintenance of sufficient drug loading is purely due to its influence on reducing the interfacial surface tension between the nano-globules.

**Table 1:** Extent of solubility of ACP in varying percentages of different types of surfactant solutions

Surfactant	Concentration (% w/v)	ACP solubility (µg/mL)
Poloxamer 188 (P188)	0.15	0.0075
	2	0.0202
Poloxamer 407 (P407)	0.15	0.2095
	2	1.7391
Tween 80 (T80)	0.15	0.0523
	2	0.1941
Polyvinyl Alcohol (PVA)	0.15	0.0137
	2	0.1087
Polyvinyl Pyrrolidone (PVP)- K30	0.15	0.7479
	2	2.2728
Gelucire 48/16	0.15	1.1844
	2	2.9627
Gelucire 44/14	0.15	1.3578
	2	2.2724

**Table 2:** Formulation batches during the development and optimization of ACP-SLNs and the influence of changes in factors (both material and process) on the PS, PDI, and %LE

Batch No	Composition	Process variable	PS (nm)/PDI	%LE
*Batches to evaluate the effect of lipid type and manufacturing method*				
F1-S25^#^	SP (125 mg); P188 (0.5% w/v) in water	Probe sonication Amplitude (25%); Time (15 min); Temperature (45 °C)	281.3/0.164	12.81
F1-H14		High shear homogenization Probe dimension (12 mm); Time (15 min); Speed (14,000 rpm)	863.6/0.747	NE^**^
F2-S25^*^	GB (200 mg); P188 (0.5% w/v) in water	Probe sonication Amplitude (25%); Time (15 min); Temperature (45 °C)	192.0/0.257	5.39
F2-H12		High shear homogenization Probe dimension (12 mm); Time (10 min); Speed (12,000 rpm)	1665.0/0.977	NE^**^
F3-S25^*^	SP:GB in 4:1 ratio; P188 (0.625% w/v) in water	Probe sonication Amplitude (25%); Time (15 min); Temperature (45 °C)	240.2/0.360	15.11
F3-H12		High shear homogenization Probe dimension (12 mm); Time (15 min); Speed (12,000 rpm); Temperature (60 °C)	823.8/0.451	NE^**^
*Batches to evaluate the effect of amount of external surfactant*				
F4-S25	SP:GB in 4:1 ratio; T80 (0.625% w/v) in water	Probe sonication Amplitude (25%); Time (15 min); Temperature (45 °C)	685.1/0.498	NE^**^
F5-S25^#^	SP:GB in 4:1 ratio; PVA (0.625% w/v) in water		320.5/0.300	8.25
F6-S25	SP:GB in 4:1 ratio; P188 (0.75% w/v) in water		402.9/0.440	15.51
F7-S25	SP:GB in 4:1 ratio; P188 (1.25% w/v) in water		515.7/0.612	17.05
*Batches to evaluate the effect of sonication parameters*				
F8-S20	SP:GB in 4:1 ratio; P188 (0.625% w/v) in water	Amplitude (20%); Time (15 min); Temperature (45 °C)	610.6/0.382	NE^**^
F9-S35		Amplitude (35%); Time (15 min); Temperature (45 °C)	442.9/0.560	8.63
F10-S25		Amplitude (25%); Time (15 min); Temperature (25 °C)	574.5/0.470	3.90
F11-S25^#^		Amplitude (25%); Time (15 min); Temperature (60 °C)	122.1/0.191	7.29
F12-S25^#^		Amplitude (25%); Time (20 min); Temperature (45 °C)	332.2/0.301	9.12
F13-S25		Amplitude (25%); Time (10 min); Temperature (45 °C)	890.5/0.770	NE^**^
*Batches to evaluate the effect of total lipid amount*				
F14-S25^*^	SP:GB in 4:1 ratio; P188 (0.625% w/v) in water	Amplitude (25%); Time (15 min); Temperature (60 °C)	217.2/0.386	14.12
F15-S25^*^	SP:GB in 2:1 ratio; P188 (0.625% w/v) in water		225.1/0.345	14.70
*Batches to evaluate the effect of incorporation of internal phase stabilizer on stability and introduction of a second sonication cycle*				
F16-S25^*^	SP:GB in 2:1 ratio; T80 (0.25% w/w) in lipid phase; P188 (0.625% w/v) in water	1st sonication cycle Amplitude (25%); Time (15 min); Temperature (60 °C) 2nd sonication cycle Amplitude (25%); Time (4 min); Temperature (25 °C)	318.5/0.336	15.31
F17-S25^*^	SP:GB in 2:1 ratio; T80 (0.5% w/w) in lipid phase; P188 (0.625% w/v) in water		262.9/0.3	12.79
F18-S25^$^	SP:GB in 2:1 ratio; T80 (0.5% w/w) in lipid phase; P188 (0.75% w/v) in water		173.4/0.361	12.09
F19-S25^$^	SP:GB in 2:1 ratio T80 (1% w/w) in lipid phase P188 (0.75% w/v) in water		219.5/0.329	15.2
F20-S25^*^	SP:GB in 2:1 ratio; T80 (1.25% w/w) in lipid phase; P188 (0.75% w/v) in water		374.2/0.567	18.86
F21-S25^$^	SP:GB in 2:1 ratio; T80 (1% w/w) in lipid phase; P188 (1% w/v) in water		213.7/0.323	17.4
F22-S25^$^	SP:GB in 7:3 ratio; T80 (1% w/w) in lipid phase; P188 (1% w/v) in water	1st sonication cycle Amplitude (25%); Time (15 min); Temperature (55 °C) 2nd sonication cycle Amplitude (25%); Time (4 min); Temperature (25 °C)	245.2/0.3	19.2

^*^Batch was stable for ~ 3 days, and ^$^batch was stable for > 5 days when stored at 2–8 °C. The increase in PS was < 2% over the 3 days. The ^#^batch was stable for ≤ 1 day when stored at 2–8 °C. PS increased due to aggregation

^**^NE = Not evaluated, as the batch does not meet the initial selection criteria of desirable PS and PDI

#### Selection of method of preparation

The formation of primary o/w microemulsions of the lipid phase (containing ACP dissolved in lipids) and aqueous phase (surfactant dissolved in water) was affected by the rate of addition of aqueous solution to the lipid phase, mixing speed, temperature (initial temperature of the individual phases before mixing and the temperature maintained during mixing of the two phases) and the duration of mixing. A higher rate of addition (8–10 mL/min), lower mixing speed (≤ 1000 rpm), and lower temperature while mixing (< 55 °C) resulted in improper dispersion of the lipid phase, where large globules of molten lipids were observed to be adhered to the walls of the preparation vessel. In addition, the duration of mixing impacted the PS of the SLNs, which eventually formed coarse globules (a reduction of ~ 1560.32 nm (in 7 min) to ~ 691.01 nm (in 15 min) when the stirring speed was maintained at 1200 rpm). At the end of 15 min, even though both the PS (691.01 nm) and PDI (0.782) were high for the primary emulsion, no traces of precipitation were observed. This ensures that for the current formulation, a solvent-free emulsion formation approach can be adapted. Furthermore, the microemulsion was subjected to high-energy particle reduction processes to form a nanoemulsion either by high shear homogenization or acoustic cavitation by probe ultrasonication. Even though a desirable range of particle sizes (281.3 nm) and drug loading capacities (12.81%) were achieved for ACP-SLNs formulated with SP alone as the solid lipid (Batch No. F1-S25), the ACP-SLNs were unstable, with visible aggregation when stored at 2–8 °C. The possible reasons for this increase may include improper lipid or surfactant concentrations, excessive sonication leading to destruction of structural integrity, thereby promoting aggregation, or improper temperatures during processing [26]. Further trials performed using GB as the only solid lipid (Batch No. F2-S25) resulted in stable SLNs. A literature survey revealed that GB can provide a more structured matrix and better aggregation resistance to temperature fluctuations, thereby helping maintain the structural stability of SLNs [27]. However, the %LE for batch no. F2–S25 was lower, as GB was able to accommodate lower amount of ACP (as shown in Fig. 1) than SP. Hence, to achieve a desirable stability and sufficient %LE , combinations of SP and GB were used as solid lipids for further trials.

Additionally, it was evident from batches F1-H14, F2-H12, and F3-H12 (Table 2) that greater PS and PDI values were obtained when high shear homogenization was used as a size reduction process. Even a variation in homogenization speed, time or processing temperature failed to produce SLNs with comparable PS to those produced by probe ultra sonication. The increase in PS can be a result of excessive shear forces created by the acceleration–deceleration process during homogenization, leading to destruction of the particles, thereby widening the PS distribution. Thus, for the current formulation where softer excipients are used, ultrasonication might be a more suitable process for controlling the particle size and the particle size distribution [28]. Ultrasonication with a standard 12 mm diameter solid probe set at 25% amplitude generated mechanical vibrations of optimum intensity to reduce the PS with a narrow span. The mechanical vibrations create a pressure difference across the internal and external phases, causing collapse of the internal phase (lipid-carrying ACP) cavitational bubbles [29]. Owing to multiple implosion sites in a confined area, significant energy is released, increasing the emulsion temperature. This could adversely affect drug and excipient properties, so a vibrational cut-off was set when the temperature of the emulsion reached a maximum of 55 °C. At such a high energy level, the lipid was suspected to be present in a molten state, thereby ensuring maximum entrapment of ACP, a lipophilic molecule. Upon completion of the sonication cycle, the intermediate formulation was stirred at ambient temperature (25 ± 2 °C) to allow gradual cooling and congelation. However, within 2 h of preparation, aggregation was observed. This could be due to two reasons: the available surfactant is either unable or insufficient to completely surround the particles, leaving their uncovered surfaces viable for hydrophobic interactions and resulting in aggregation [30]. Hence, to address the stability issue, a combined strategy of material and process parameters was introduced: first, to overcome the deficiency of a surfactant, an amphiphilic surfactant, T80, was added to the lipid phase at varying percentages (0.25–1.25% w/w of the total solid mass, including the lipids and drug). Second, when the temperature (when gradual cooling was allowed) of the emulsion was 30 ± 5 °C, a second cycle of sonication (25% amplitude, sonication cut off when the liquid reached a maximum of 25 °C) was performed for 4 min to ensure gradual recrystallization of the lipid, entrapment of ACP, and reduction of the particle size. This combination of surfactant and sonication (Batches F16–S25 to F22–S25, Table 2) resulted in a stable formulation with the desired PS and PDI (Batch No. F22–S25, Table 2). The optimized formulation was reproducible (n = 6 batches with a batch volume of 50 mL and n = 3 batches with a batch volume of 100 mL, PS = 245.2 ± 10.5 nm and PDI = 0.301 ± 0.04) and stable for up to 7 days of preparation (PS = 296.9 nm and PDI = 0.498) with no observance of precipitation when stored at 2–8 °C.

### Physical characterization of the ACP-SLNs

#### Size and morphology of ACP-SLNs

**Fig. 2:**
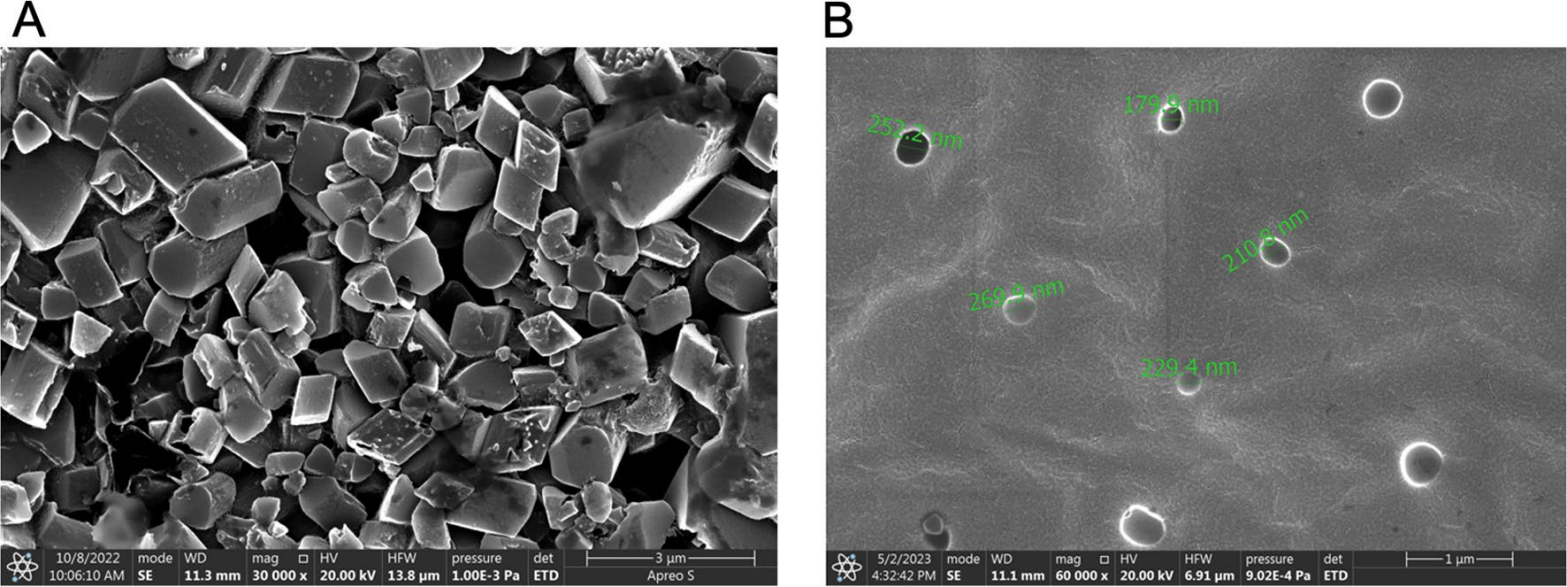
SEM image depicting the morphology and particle size of ACP bulk drug (**A**), and ACP-SLNs (**B**)

The influence of various material attributes and process parameters on the particle size and PDI during the development of SLNs is tabulated in Table 2. For the batches manufactured via optimized methods parameters (both material and process parameters), a PS of 245.2 ± 10.5 nm with a PDI of 0.301 ± 0.04 was observed for the freshly prepared ACP-SLNs nanosuspension, whereas a PS of 251.8 ± 5.7 nm with a PDI of 0.310 ± 0.10 was observed for reconstituted freeze-dried ACP-SLNs. A lower PDI and CV (< 4.3%) for PS represents a narrow particle size distribution, in turn emphasizing the efficiency of the manufacturing process to achieve uniform PS for the SLNs. As both the internal and the external phase stabilizers are non-ionic, they provide stearic stabilization to the SLNs both during preparation and storage. The zeta potential values of the freshly prepared ACP-SLNs nanosuspension and reconstituted freeze-dried ACP-SLNs powder were -2.10 ± 1.8 mV and -5.29 ± 2.02 mV, respectively. Morphological evaluation via SEM (Fig. 2) revealed that the formulated SLNs were spherical in shape with smooth surfaces. Additionally, the PS values recorded during SEM were strongly correlated with those obtained from the zeta sizer.

#### Thermal analysis of ACP-SLNs

**Fig. 3:**
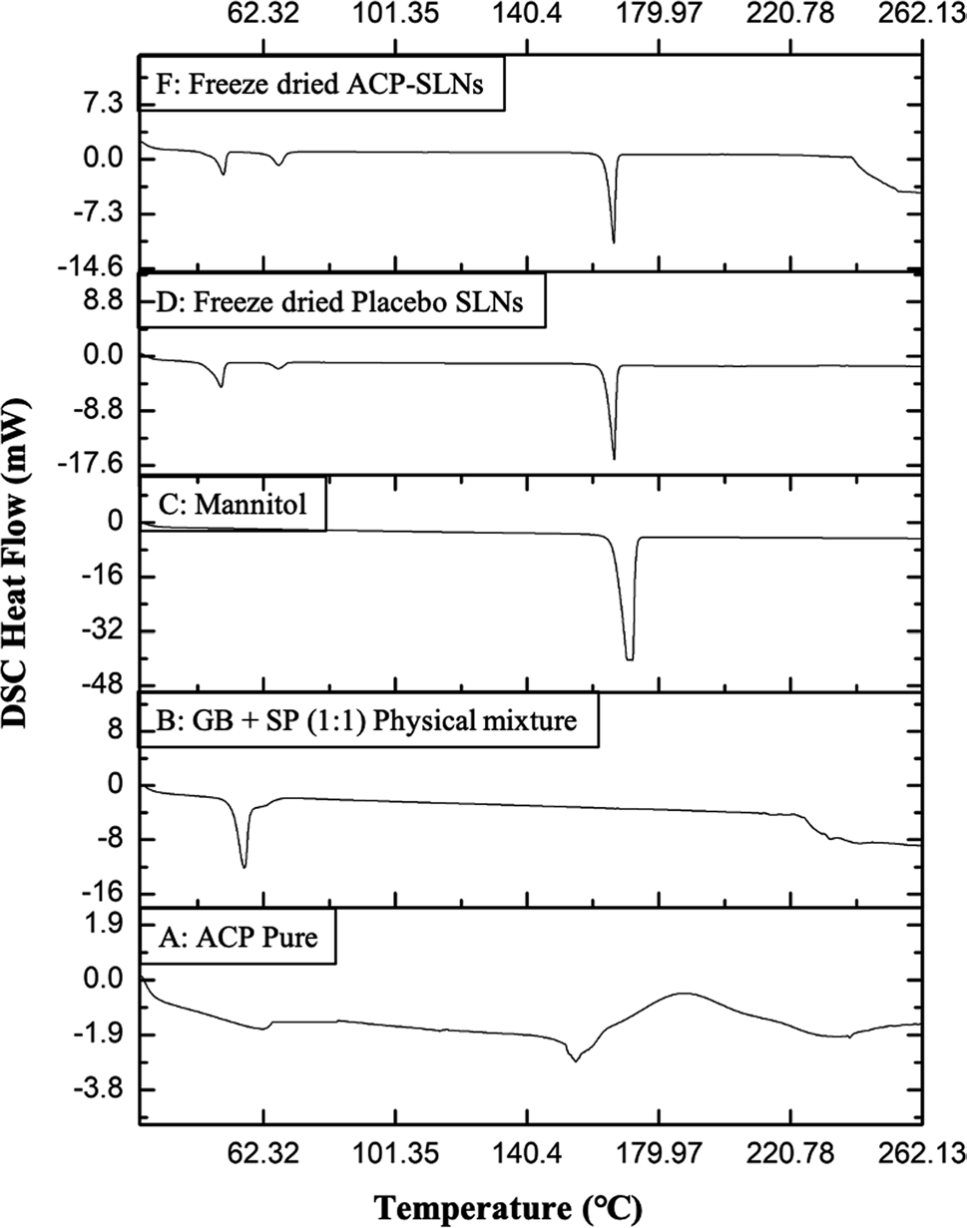
DSC overlay of Pure ACP (**A**); GB and SP (1:1) physical mixture (**B**); mannitol (cryoprotectant) (**C**); freeze-dried placebo SLNs (**D**); and ACP-SLNs (**F**). In the thermograms, endotherms and exotherms are represented as downwards and upwards peaks, respectively

Figure 3 shows the overlay of DSC thermograms of bulk ACP; a physical mixture of GB and SP (1:1); bulk mannitol (cryoprotectant); freeze-dried placebo SLNs; and freeze-dried ACP-SLNs. Twohe characteristic endothermic (~ 159 °C) and exothermic peaks (~ 200 °C) were observed in case of bulk ACP (Fig. 3A). The endothermic peaks at ~ 55 °C to 63 °C correspond to the melting of the physical mixture of the solid lipids (GB and SP) (Fig. 3B) whereas, the prominent endothermic peak at ~ 170 °C corresponds to the melting of mannitol (Fig. 3C). The placebo SLNs exhibited melting endotherm similar to that of mannitol (Fig. 3D). In case of freeze-dried ACP-SLNs, the characteristic peaks of pure ACP were not observed (Fig. 3F), indicating that ACP was entrapped either in its molecular form or amorphous form in the nanoparticles. The melting endotherm in Fig. 3F also corresponds to that of the cryoprotectant.

#### P-XRD of ACP-SLNs

**Fig. 4:**
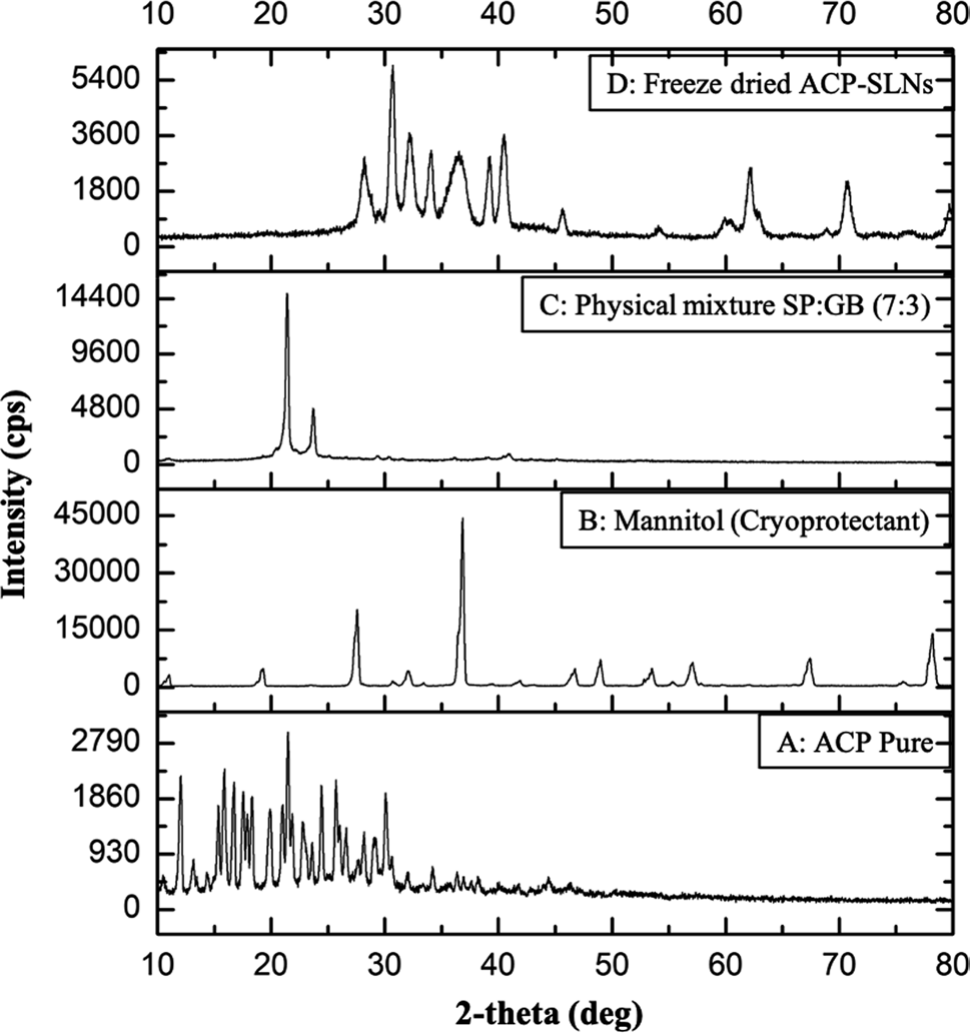
P-XRD overlay of Pure ACP (**A**); mannitol (cryoprotectant) (**B**); SP:GB (7:3) physical mixture (**C**); and freeze-dried ACP-SLNs (**D**)

Figure 4 demonstrates the comparative analysis between the final optimized freeze-dried product and the various formulation components. For pure ACP (Fig. 4A) intense characteristic peaks at 2-θ values of 12.03°; 16.71°; 24.43°; and 25.69° represented its crystalline property. For mannitol and physical mixture of the solid lipids, intense peaks were observed (Fig. 4B and C) at 27.25°, 36°; and 21.38°, 23.72°, respectively representing their crystalline nature. Figure 4D represented the diffractogram of freeze-dried ACP-SLNs which was devoid of the characteristic peaks observed for pure ACP inferring that ACP was present in the molecular or amorphous state in the current formulation.

### Loading and encapsulation efficiency of ACP-SLNs

The final optimized batch of ACP-SLNs (batch no. F22-S25), upon evaluating the batch-to-batch reproducibility of the 6 batches (n = 6), had %LE and %EE values of 18.70 ± 1.78% and 53.44 ± 4.28%, respectively. The %LE and %EE were affected by the total amount of lipids, the affinity of the drug to the lipids and the amount of internal phase stabilizer. On the basis of the results of the solubility studies (Sect. 4.1.1), ACP has good affinity for the lipids (> 80 mg in 200 mg of SP and ~ 40 mg in 200 mg of GB) used in the preparation of SLNs. In addition, the solubility of ACP in 1% w/v solutions of T80 and P188 was less than 0.1 µg/mL. Such a low solubility in the surfactant solution (which formed the continuous phase) decreases the chances of drug partitioning from the lipid phase (internal phase) into the continuous phase during the manufacturing process. This in turn increases the %LE and %EE in the SLNs. Finally, the imperfections in the internal structure of the SLNs during the congealing of the lipid globules into the solid lipid nanoparticles play a role in the %LE and %EE of the drug in the SLNs. Western and co-workers (1997) experimentally demonstrated that, compared to single lipids, acylglycerol mixtures offer lower crystallinity in SLN formulations, thereby providing better drug distribution within the matrix and higher %LE and %EE [31]. The current SLN system contains a mixture of SP and GB along with T80, providing an imperfect lattice arrangement of the lipids in the formation of lipid nanoparticles, resulting in sufficient loading and entrapment of ACP.

### In vitro dissolution studies

The inherent hydrophobicity of ACP results in a solubility profile that is dependent on pH. Pepin et al. (2019) reported that the solubility of ACP was 29.2 mg/mL in 0.1 N HCl (pH = 1); 3.73 mg/mL in 0.01 N HCl (pH = 2); 0.159 mg/mL in acetate or citrate buffer (pH = 4.5); and subsequently decreased to 0.0523 mg/mL in pH 6.8 phosphate buffer solution [32]. It is very important to maintain sink condition during the in vitro dissolution study of any drug product. To maintain sink condition in the in vitro dissolution studies of hydrophobic drugs, various strategies can be employed, including, addition of surfactant; maintaining large volumes of dissolution media; addition of co-solvents; and use of a different dissolution apparatus like a flow through cell etc. [33]. Of all the above strategies, addition of surfactants (either ionic or non-ionic) is the most commonly employed strategy. SDS, an anionic surfactant, is used to improve the wettability and saturation solubility of hydrophobic drugs in aqueous media. Researchers have used SDS in the range of 0.2 to 20% w/v to maintain the sink condition in the in vitro dissolution studies of formulations containing hydrophobic molecules [33–35]. Thus, in order to achieve a sink condition, 0.5% w/v SDS was added in buffer systems having a pH of 4.5 and higher. The saturation solubility of ACP in pH 4.5; 6.8; and 7.4 in the presence of 0.5% w/v SDS was ≥ 0.77 mg/mL. Further, to maintain the sink condition, volumes of the dissolution media were taken such that the media can accommodate more than 3-times the saturation solubility concentration of ACP when the entire drug dose present in the drug product is release/dissolved. Based on the above criteria, we have determined the minimum volume of pH 4.5, pH 6.8 and pH 7.4 (containing 0.5% w/v SDS) required to dissolve the entire drug dose administered in the formulation to be at least 130 mL. The volumes should also be comparable to the physiological volume of fluids present at the relevant sites (stomach, in fed condition (pH 4.5), ileum (pH 6.8) and systemic circulation (pH 7.4)). Additionally, Pepin and his co-workers have demonstrated the behaviour of ACP with SDS in aqueous dissolution medium [32]. 

**Fig. 5:**
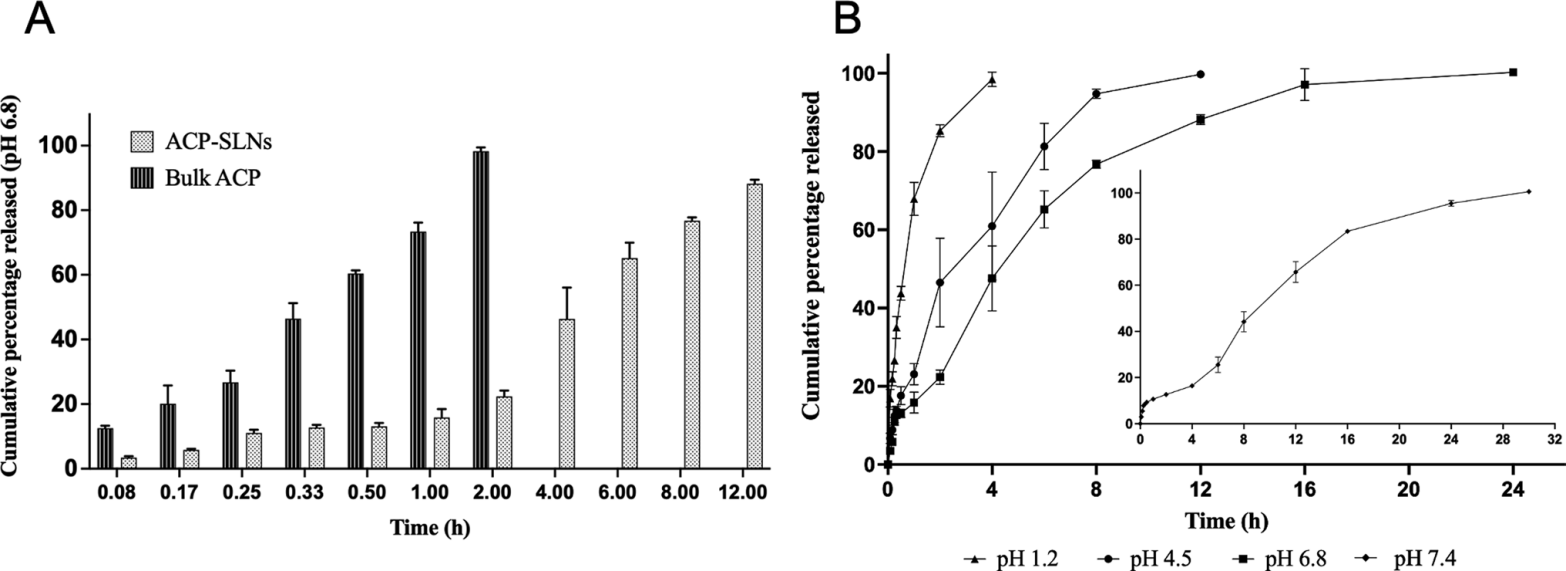
Comparative analysis of In vitro ACP release profiles- Cumulative percentage ACP released/dissolved in pH 6.8 (**A**), and cumulative percentage ACP released from the freeze-dried ACP-SLNs in different pH conditions (**B**)

Figure 5A shows that in the presence of 0.5% w/v SDS at pH 6.8, more than 80% of the ACP dissolved within 2 h. However, when ACP is encapsulated in SLN matrix, it results in sustained drug release, with the potential to improve the in vivo absorption. Figure 5B shows that ACP-SLNs exhibit a biphasic release profile across various pH conditions. Within 10–15 min of dispersion of the freeze-dried ACP-SLNs in the dissolution media, at least 10% of the drug is released regardless of the pH of the medium. This initial rapid release is attributed to the SLN preparation process, where a considerable amount of ACP was disposed off on the outer layer as the temperature of the emulsion gradually decreased, lipids started congealing, and the solubility of the ACP in the aqueous medium containing P188 (1% w/v) decreased [36]. At pH 1.2, ACP from the SLNs were released faster and dissolved within 4 h. As the pH increased to 4.5, 6.8, and 7.4, the release rate decreased, with more than 90% of the drug being released after 8, 16, and 24 h, respectively. Notably, no precipitation was observed at pH values greater than 6, both during the initial study period and when the maximum amount of ACP was released. These findings suggest that ACP-SLNs can effectively increase drug solubility at intestinal and systemic pH levels, increasing the likelihood of efficient systemic absorption.

Release rate kinetics, analysed via a model-dependent method, indicated that SLNs follow a square root or Higuchi model with a regression coefficient (r^2^) greater than 0.96. This finding suggests that ACP release primarily occurs through unidirectional diffusion from the lipophilic matrix.

### Stability analysis of ACP-SLNs

**Fig. 6:**
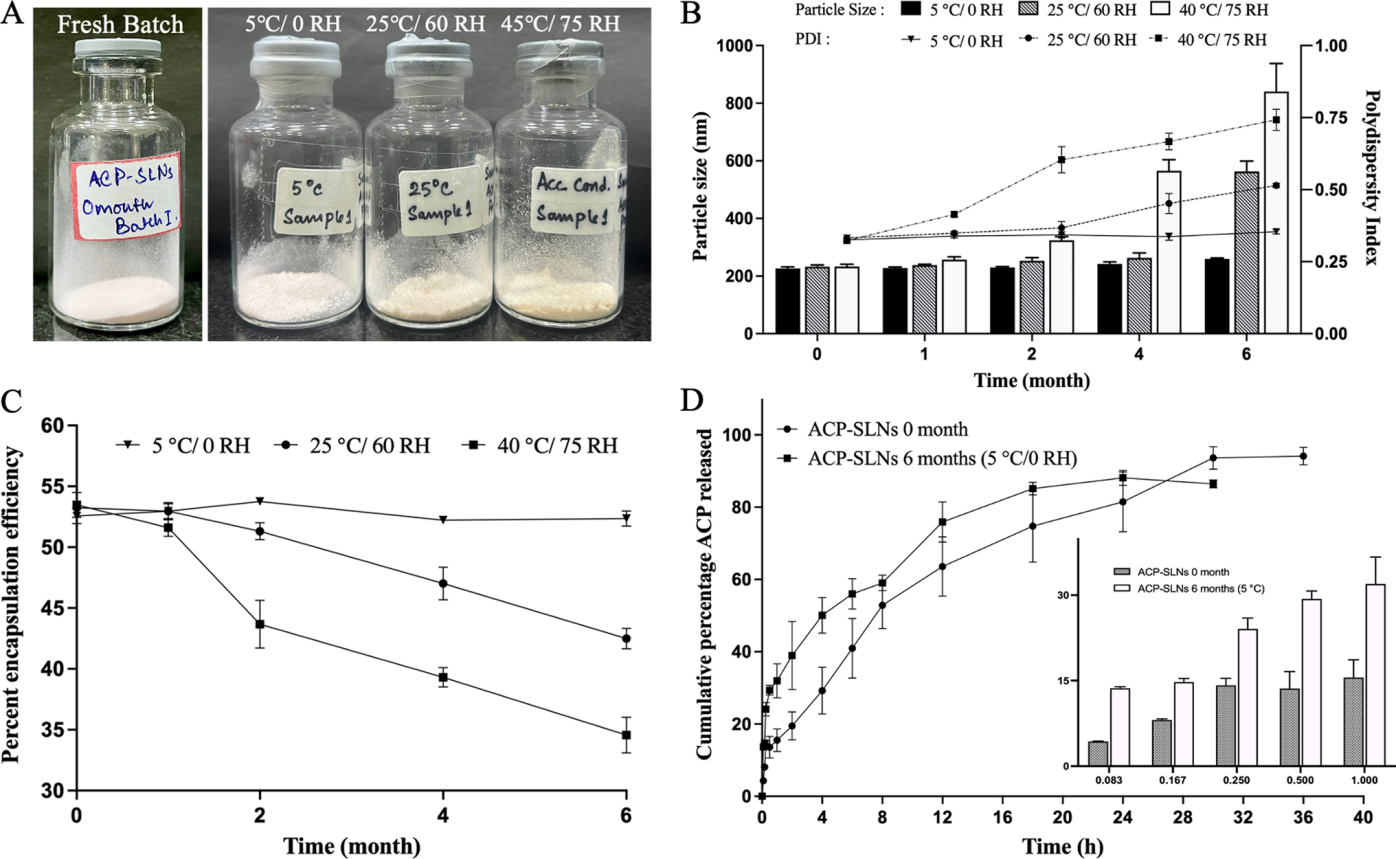
Stability analysis of freeze-dried ACP-SLNs in three different storage conditions- physical appearance of the freshly prepared sample and the samples stored for 6 months in three different storage conditions (**A**); changes in PS and PDI over 6 months of storage in three different storage conditions (**B**); chages in encapsulation efficiency of samples over 6 months of storage in three different storage conditions (**C**) and comparative dissolution profiles, in pH 7.4, of freshly prepared formulation and formulation stored for 6 months at 5 °C (**D**)

Storage stability analysis was performed only for the freeze-dried powder of ACP-SLNs, as the formulation is intended to be available in a powder state rather than a liquid form (aqueous nanosuspension of ACP-SLNs). Upon storage at three storage conditions, 45 ± 2 °C/75 ± 5% RH, 25 ± 2 °C/60 ± 5% RH and 5 ± 2 °C over 6 months, the physical appearance of the freeze-dried ACP-SLNs was retained only for the formulation samples stored at 5 ± 2 °C (Fig. 6A). For those stored at 25 ± 2 °C/60 ± 5% RH, the appearance was intact until 4 months, whereas the flowability was similar until 6 months. The samples stored at 45 ± 2 °C/75 ± 5% RH lost both appearance and flowability by 6 months (Fig. 6A). This change in physical appearance may be due to oxidation of the lipids in the presence of elevated temperature and humidity. The changes in the PS (along with the PDI) and %EE of the ACP-SLNs stored under the three storage conditions are depicted in Fig. 6B and 6C, respectively. The particle size of the SLNs, when stored at 25 ± 2 °C/60 ± 5% RH, was intact till 4 months proving the aforementioned observation regarding the physical appearance of the same. While, a gradual increment in the particle and PDI was observed for samples stored in 45 ± 2 °C/75 ± 5% RH. The reduction in the %EE of the ACP-SLNs at 45 ± 2 °C/75 ± 5% RH and 25 ± 2 °C/60 ± 5% RH storage conditions over time could be due to alterations in the crystal arrangement or temperature-induced softening of the lipid. Due to the softening of lipid matrix, the entrapped ACP can diffuse out of the matrix, reducing the %EE. As shown in Fig. 6B and C, samples stored at 5 ± 2 °C presented no differences in any of the three evaluation parameters, PS, PDI or %EE, over the 6-month period. At 5 ± 2 °C, the added cryoprotectant during freeze-drying (2% w/v mannitol), due to its vitrification process, provides a rigid scaffold structure which prevented the movement of particles and thereby any possible aggregation of the particles. In addition, at 5 ± 2 °C, due to the solid crystal structure of the lipids the diffusion of the drug from the lipid matrix of NLCs was prevented and the %EE remained unchanged during the 6-month period. Therefore, only the samples stored at 5 ± 2 °C for a 6-month period were evaluated for drug release studies at pH 7.4 ± 0.2 (as described in Sect. 3.3.4). The dissolution profile of the stable sample (Fig. 6D) was compared with that of the freshly formulated ACP-SLNs. Drug release was relatively greater, at least in the initial period (until 6 h), for the samples stored for 6 months than for the freshly prepared formulation. This could be due to changes in the lipid lattice structure of the SLNs and, thereby, the distribution of the entrapped drug in the SLN matrix. The deviations in the dissolution profile were also reflected in the decrease in the similarity index (f2), which was 36.72%.

### Haemolytic assay

**Fig. 7:**
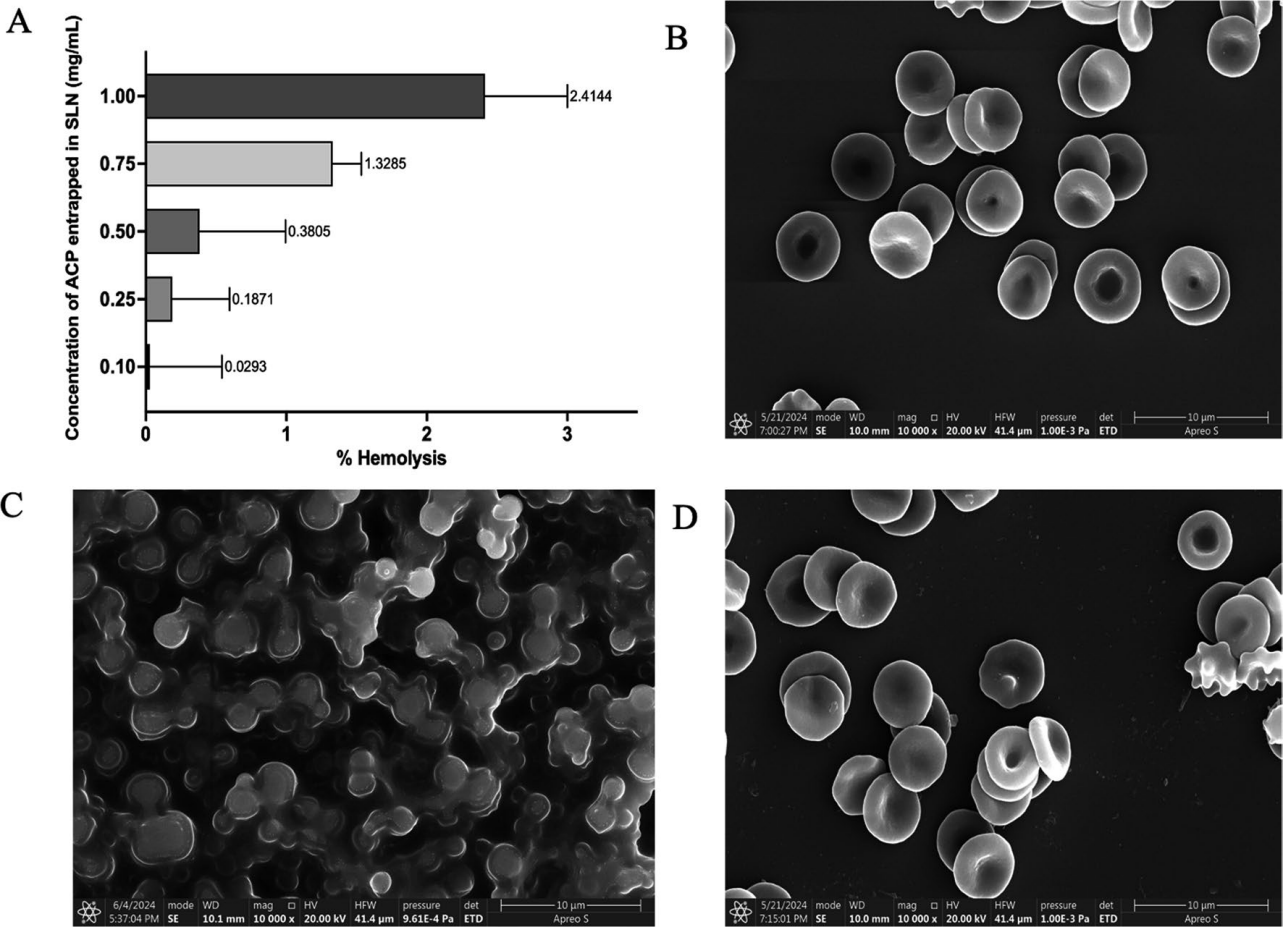
Percentage hemolysis assay by optical absorbance for ACP-SLNs containing different concentrations (**A**); SEM images depicting morphological changes between healthy (negative control) (**B**), lysed (positive control), (**C**) and ACP-SLNs treated RBCs (**D**)

Figure 7A shows the quantitative estimation of haemolysis caused by incubating the blood samples with ACP-SLNs nanosuspensions containing different effective concentrations of ACP. A maximum of 2.93% lysis was observed for the cells incubated with the ACP-SLNs nanosuspension containing ACP at an effective concentration of 1 mg/mL. Figures 7B, C and D show images depicting the visual changes in the RBCs subjected to different treatments in the study. The RBCs in Fig. 7B represent the healthy cells, the ones treated with saline solution (negative control). Figure 7C shows morphology of RBCs treated with 1% v/v Triton X (positive control), where the RBCs completely lysed. Figure 7D presents SEM images (magnified at 2000–2500 ×) of RBCs at T_max_ (45 min) after the administration of ACP-SLNs suspension (at a dose of 30 mg/kg) to Wistar rats exhibiting morphological features similar to that of the negative control. The results of this study indicate that even at the likely maximum concentration of ACP in blood, after the oral administration of ACP-SLNs, there was no significant effect on the integrity of the RBCs. These findings suggest that the ACP-SLNs are free from any haemolytic toxicity.

### Pharmacokinetic studies of ACP-SLNs

#### Intravenous bolus and oral pharmacokinetic studies of ACP-SLNs

**Fig. 8:**
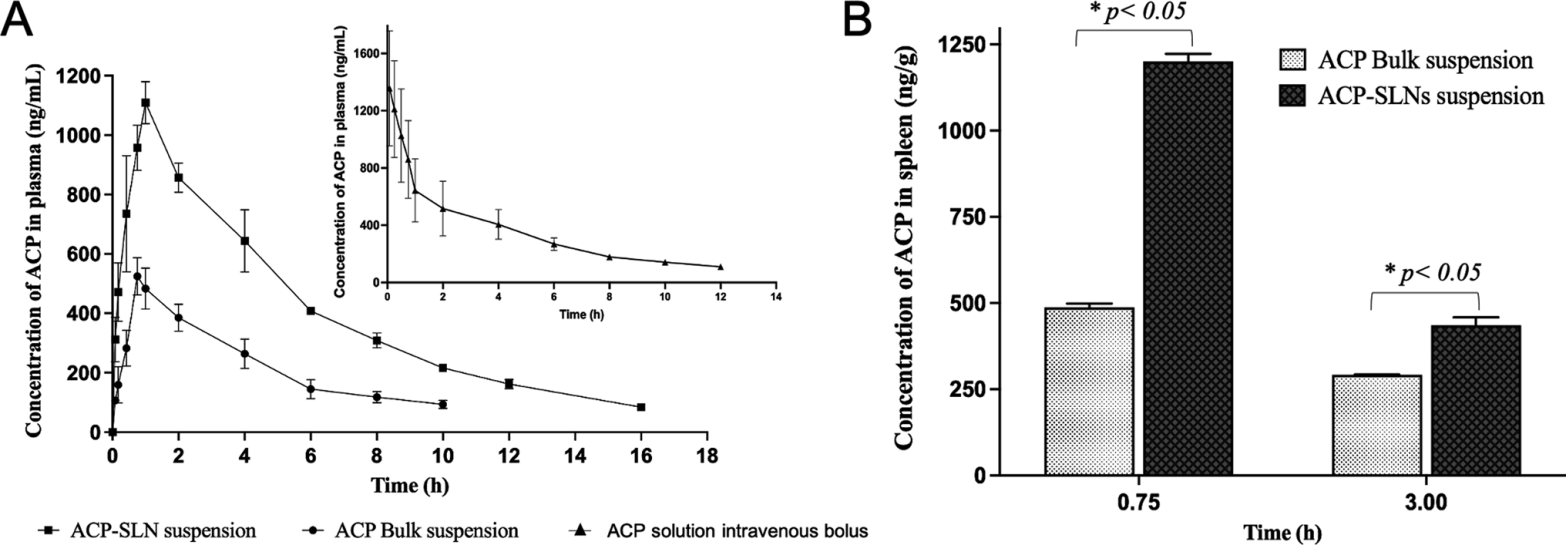
Comparative analysis of in vivo PK profile obtained from oral administration of ACP bulk suspension and ACP-SLNs suspension to male wistar rats (n = 3) (**A**); and comparison between the distribution of ACP towards spleen for ACP bulk suspension and ACP-SLNs suspension administered orally to male Wistar rats (n = 2) (**B**)

The comparative oral pharmacokinetic profiles of aqueous suspension of bulk ACP and ACP-SLNs are presented in Fig. 8A. The comparative pharmacokinetic parameters of both formulations are given in Table 3. ACP-SLNs suspension produced significantly greater systemic exposure to ACP (in terms of both C_max_ ; AUC_0-tlast_; and AUC_0-∞_) than aqueous suspension of bulk ACP. The AUC_0-tlast_ for ACP-SLNs suspension (6383.67 ± 215.70 h $\times$ ng/mL) was 2.29 times (*p* < 0.001) greater than that for the aqueous suspension of ACP (2299.62 ± 173.49 h $\times$ ng/mL). The C_max_ of ACP-SLNs suspension (1109.24 ± 70.90 ng/mL) was 1.98 times (*p* < 0.001) greater than that of the aqueous suspension of ACP (561.19 ± 4.62 ng/mL). The improved pharmacokinetic parameters arise from several factors. First, the nanoscale size of SLNs provides a larger surface area, enhancing drug distribution into the luminal fluid. Despite ACP’s low solubility at intestinal pH (≥ pH 6.8), the SLNs maintain drug release in a dissolved state due to presence of lipids and surfactants that facilitate micelle or mixed-micelle formation (with endogenous bile salts). Additionally, the nano size allows SLNs to be directly taken up by endocytosis, bypassing dissolution and absorption processes. Once in systemic circulation, ACP remains at pH 7.4 ± 2, as shown by in vitro dissolution studies. Moreover, ACP within SLNs is shielded from extensive metabolism by the CYP3A4 or P-gp efflux system, reducing metabolic loss. Further, at a given time a high concentration of dissolved ACP is sufficient to saturate these enzyme systems, allowing more drug to be absorbed through the luminal wall. Finally, SLNs are also taken up by lymphatic pathways, further minimizing chances of metabolism and potentially enhancing systemic drug exposure.

**Table 3:** PK parameters obtained after analysis of the plasma concentrations of the ACP bulk, ACP-SLNs (in healthy rats), and ACP-SLNs (in CHX-treated rats) suspension

Parameters		Values		
		ACP bulk suspension	ACP-SLNs suspension (- CHX)	ACP-SLNs suspension (+ CHX)
T_max_	*h*	0.75	1	1
C_max_	*ng/mL*	561.19 ± 4.62	1109.24 ± 70.90	810.35 ± 9.10
C_max_/Dose	*ng/mL/mg*	77.94 ± 0.64	147.90 ± 9.45	122.78 ± 1.38
AUC_0-tlast_	h*ng/mL	2299.62 ± 173.49	6383.67 ± 215.70	3442.33 ± 78.74
AUC_0-∞_	h*ng/mL	3019.88 ± 47.66	6912.20 ± 191.50	3933.44 ± 86.31
MRT_0-tlast_	*h*	3.50 ± 0.15	6.22 ± 0.21	5.71 ± 0.28
F^#^	*-*	20.16 ± 3.17	46.16 ± 7.37	26.28 ± 4.44
Cl/F	*mL/h*	2384.60 ± 37.94	1085.59 ± 29.70	1678.46 ± 36.84

All the values are reported as the means ± SDs (n = 3), except for T_max,_ which is expressed as the median of 3 independent determinations

^#^The absolute bio-availabilities of both bulk ACP and ACP-SLNs suspension were determined via the formula $F = \left[ {\frac{{\left( {AUC_{0 - \infty } } \right)_{Oral} }}{{\left( {AUC_{0 - \infty } } \right)_{IV} }} \times \frac{{Dose_{IV} }}{{Dose_{Oral} }}} \right] \times 100$

The mean residence time (MRT_0-tlast_) of the ACP-SLNs suspension (6.22 ± 0.21 h) was significantly greater (*p* < *0.01*) than that of the aqueous suspension of bulk ACP. These findings suggest that the plasma drug concentrations of ACP-SLNs suspension were sustained than those of aqueous suspension of bulk ACP. This can also be attributed to the lower clearance of ACP-SLNs suspension (1085.59 ± 29.70 mL/h) than of aqueous suspension of bulk ACP (2384.60 ± 37.94 mL/h). The absolute bioavailability of ACP-SLNs suspension was 46.16 ± 7.37%, whereas that of aqueous suspension of bulk ACP was 20.16 ± 3.17%.

#### Distribution of the ACP to the Spleen

The spleen is an important target organ in the treatment of CLL. A greater distribution of drugs to the spleen can lead to better therapeutic outcomes in the treatment of CLL. Figure 8B presents the concentration of ACP in the spleen at 0.75 h and 3 h for aqueous suspensions of bulk ACP and ACP-SLNs. At both time points, ACP-SLNs suspension (C_0.7 h_ = 1200.65 ± 22.39 ng/g and C_3h_ = 435.17 ± 29.2 ng/g) resulted in significantly greater (*p* < *0.01*) drug distribution to the spleen than aqueous suspension of bulk ACP (C_0.7 h_ = 487.23 ± 11.29 ng/g and C_3h_ = 291.02 ± 1.28 ng/g). This could be due to the passive targeting of the nanoparticles to the spleen and to the longer residence time of the drug, resulting in greater drug distribution to the spleen.

#### Lymphatic uptake studies of ACP-SLNs

**Fig. 9:**
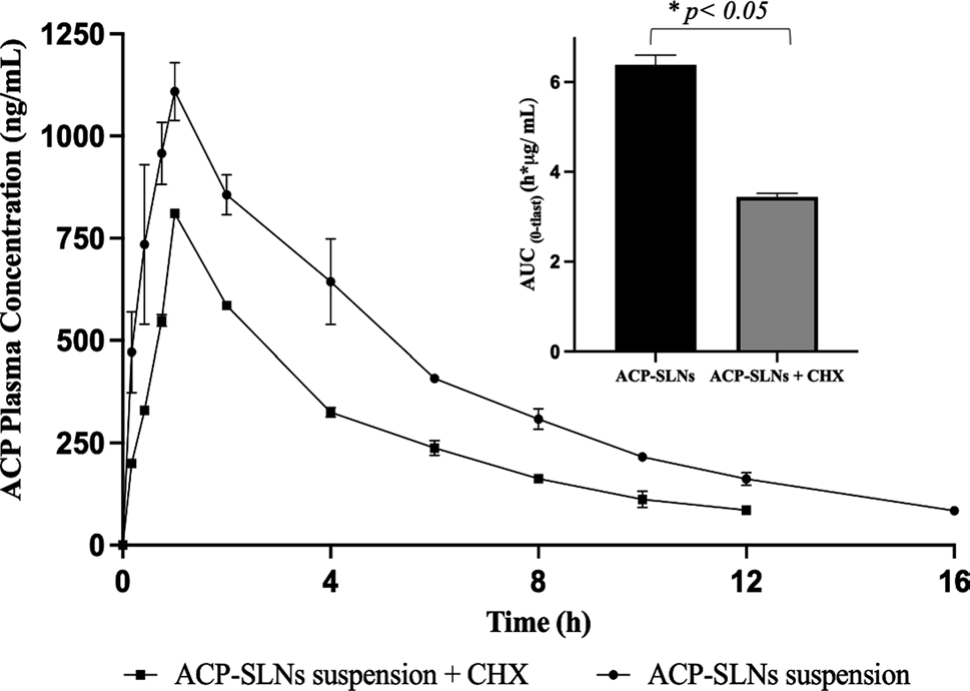
Overlay of in vivo PK profile upon administration of ACP-SLNs suspension in healthy and CHX treated rats (*n* = 3). Comparative analysis of the AUC(*0-T*last) between both the treatment groups (nested figure)

According to previous reports, SLNs can be taken up by the lymphatic system via two mechanisms: lymphoid follicles (M-cells and gut-associated lymphoid tissue (GALT)) associated with Peyer’s patches and the partitioning of fatty acids and monoglycerides into triglycerides, which form the core of lipoproteins or chylomicrons [19, 20, 37]. ACP is a lipophilic molecule with a high affinity towards the lipid core and tends to be taken up by the lymphatic system. Figure 8 depicts the overall decrease in the plasma concentration of ACP in the presence of CHX. According to the reports of Dahan and Hoffman (2005), CHX primarily operates as a protein synthesis inhibitor that is selective for inhibiting the formation of chylomicrons and, in turn, the flow of lymph without hampering the active and/or passive uptake pathways present in the body (in this case, the intestinal lumen) [38]. The study revealed a marked decrease in the C_max_ and AUC by 26.73 ± 5.08% and 46.01 ± 3% (nested plot in Fig. 9), respectively (p < 0.05), emphasizing the possibility that the improved pharmacokinetic profile of the ACP-SLNs suspension in comparison with that of the conventional ACP suspension is hugely influenced by the lymphatic uptake mechanism.

## Conclusion

Acalabrutinib-loaded solid lipid nanoparticles (ACP-SLNs) were successfully prepared using glyceryl di-behenate and stearyl palmitate through solvent-free hot emulsification followed by double sonication. The optimization of the excipients and manufacturing parameters achieved a loading efficiency of 18.7% and an entrapment efficiency of 53.4%. The ACP-SLNs had a particle size of < 300 nm and remained stable for 6 months under refrigeration. Drug release from ACP-SLNs was slow and sustained for more than 24 h. Compared to the conventional aqueous suspension, ACP-SLNs suspension presented a 2.29-fold increase in absolute oral bioavailability and achieved sustained plasma drug concentrations, with greater drug distribution towards the spleen. In cycloheximide-treated rats, ACP-SLNs suspension demonstrated a significant lymphatic uptake highlighting the benefits of lipid based carrier systems in enhancing the systemic exposure of molecules possessing several biopharmaceutical challenges. Overall, ACP-SLNs offer improved solubility and dissolution behaviour, oral bioavailability, prolonged in vivo residence time, and enhanced spleen distribution, potentially leading to better clinical outcomes for CLL treatment than conventional formulations do.

## Data Availability

The datasets generated during and/or analysed during the current study will be available from the corresponding author, Punna Rao Ravi (rpunnarao@hyderabad.bits-pilani.ac.in) on a reasonable request.
